# Lipopolysaccharide-Mediated Effects of the Microbiota on Sleep and Body Temperature

**DOI:** 10.21203/rs.3.rs-3995260/v1

**Published:** 2024-03-05

**Authors:** Eva Szentirmai, Katelin Buckley, Ashley R. Massie, Levente Kapas

**Affiliations:** Washington State University Spokane; Washington State University Spokane; Washington State University Spokane; Washington State University Spokane

**Keywords:** endotoxin, lipopolysaccharide, microbiota, sleep, body temperature, fever, prostaglandins, liver, portal vein, hepatoportal sensors

## Abstract

**Background:**

Recent research suggests that microbial molecules translocated from the intestinal lumen into the host’s internal environment may play a role in various physiological functions, including sleep. Previously, we identified that butyrate, a short-chain fatty acid, produced by intestinal bacteria, and lipoteichoic acid, a cell wall component of gram-positive bacteria induce sleep when their naturally occurring translocation is mimicked by direct delivery into the portal vein. Building upon these findings, we aimed to explore the sleep signaling potential of intraportally administered lipopolysaccharide, a primary component of gram-negative bacterial cell walls, in rats.

**Results:**

Low dose of lipopolysaccharide (1 μg/kg) increased sleep duration and prolonged fever, without affecting systemic lipopolysaccharide levels. Interestingly, administering LPS systemically outside the portal region at a dose 20 times higher did not affect sleep, indicating a localized sensitivity within the hepatoportal region, encompassing the portal vein and liver, for the sleep and febrile effects of lipopolysaccharide. Furthermore, both the sleep- and fever-inducing effects of LPS were inhibited by indomethacin, a prostaglandin synthesis inhibitor, and replicated by intraportal administration of prostaglandin E_2_ or arachidonic acid, suggesting the involvement of the prostaglandin system in mediating these actions.

**Conclusions:**

These findings underscore the dynamic influence of lipopolysaccharide in the hepatoportal region on sleep and fever mechanisms, contributing to a complex microbial molecular assembly that orchestrates communication between the intestinal microbiota and brain. Lipopolysaccharide is a physiological component of plasma in both the portal and extra-portal circulation, with its levels rising in response to everyday challenges like high-fat meals, moderate alcohol intake, sleep loss and psychological stress. The increased translocation of lipopolysaccharide under such conditions may account for their physiological impact in daily life, highlighting the intricate interplay between microbial molecules and host physiology.

## Introduction

The mounting evidence on the impact of the intestinal microbiota on various host physiological responses has sparked interest in understanding the role of microbial molecules in influencing brain functions. The microbiota is implicated in fundamental brain processes such as neuroregeneration, behavior, memory and various nervous system pathologies (reviewed in Ma et al., 2019). For example, there is growing evidence highlighting the relationship between the microbiota and conditions such as Alzheimer’s disease, Parkinson’s disease, depression and anxiety.

A dynamic bidirectional relationship connects the brain’s sleep mechanisms with the gut flora. Depletion of the intestinal microbiota results in a substantial reduction in sleep duration, underscoring the role of gut flora as a source of sleep-inducing signals (Brown et al., 1990; Millican et al., 2018; Ogawa et al.,2020). Conversely, disruptions to normal sleep-wake rhythms, such as circadian disturbances, acute sleep loss or chronic sleep fragmentation influence the composition of the microbiota (Wang et al., 2021; Poroyko et al., 2016; Benedict et al., 2016; Voigt et al., 2016). Microbial molecules have long been recognized for their ability to induce sleep when administered systemically or directly into the brain. Their effects, such as those induced by high doses of bacterial cell wall products like peptidoglycan fragments (Krueger et al., 1982), lipoteichoic acid (LTA) (Szentirmai et al., 2021), and lipopolysaccharide (LPS) (Lancel et al., 1995; Kapas et al., 1998; Opp and Toth, 1998; Szentirmai and Krueger, 2014), were traditionally interpreted within the context of systemic inflammation and sickness behavior/syndrome, given their resemblance to signs and symptoms of systemic bacterial infections.

However, there is a shift in perspective toward recognizing these microbial components as inherent, physiological elements in the host’s internal environment, constituting a normal physiological presence in plasma (Nolan, 1981; Wiedermann et al., 1999; Goto et al., 1994). These microbial metabolites enter the circulation through translocation from the intestinal lumen into the portal vein, which drains venous blood rich in absorbed nutrients and microbial molecules from the intestinal system to the liver (Guerville and Boudry, 2016). LPS, a major component of the gram-negative bacterial cell wall, is released from dividing and dead bacteria in the intestinal lumen and translocates in significant amounts via a toll-like receptor 4 (TLR4)-mediated mechanism (Cetin et al., 2004). It is present in the portal and extra-portal systemic circulation under healthy conditions reaching the liver through the portal inflow, where the liver serves as an LPS sink, sequestering circulating LPS (Fox et al., 1990).

The translocation of LPS and other microbial molecules from the intestinal lumen is regulated by the intestinal barrier (Guerville and Boudry, 2016). Everyday challenges that humans encounter have a major impact on the integrity of the intestinal barrier and thus on the circulating levels of microbial molecules. For example, a single fat-rich meal Erridge et al., 2007, moderate amounts of alcohol intake (Ferrier et al., 2006; Sturm et al., 2021), sleep loss (Wang et al., 2021; Li et al., 2024) or mild psychological stress (Vanuytsel et al., 2013; Santos et al., 2000) impair this barrier, resulting in elevated levels of circulating LPS. Additionally, the diurnal fluctuation in microbiota composition, bacterial adherence to the intestinal mucosa, metabolite production and circulating levels of LPS further underscore the dynamic nature of microbiota-host interactions (Cani et al., 2007; Zarrinpar et al., 2014; Thaiss et al., 2016; Segers et al., 2018).

Recent evidence supports the hypothesis that translocated microbial molecules might exert a modulatory influence on brain functions, particularly sleep. For instance, butyrate, a short-chain fatty acid produced by intestinal bacteria, and LTA, a gram-positive bacterial cell wall component, induce sleep acting within the hepatoportal region (Szentirmai et al., 2021; Szentirmai et al., 2019). The identification of a sleep-inducing sensory mechanism in this region led us to propose that translocated LPS from the intestinal microbiota plays a role in modulating sleep through the same or comparable mechanism.

We report that, consistent with this hypothesis, intraportal administration of LPS, at doses that do not alter plasma levels in the extra-portal circulation, effectively induces sleep and fever. These effects are suppressed by indomethacin, a prostaglandin synthesis inhibitor, and can be replicated by intraportal administration of prostaglandin E_2_ (PGE_2_) or arachidonic acid, a precursor of prostaglandin synthesis. These findings strongly suggest that LPS in the portal circulation dynamically influences mechanisms related to sleep and fever, contributing to a microbial molecular assembly that facilitates communication between the intestinal microbiota and brain functions.

## Methods

### Animals

All animal procedures were conducted in accordance with the recommendations outlined in the Guide for the Care and Use of Laboratory Animals by the National Institutes of Health. The animal care and experimental protocols adhered to the guidelines established by the Association for Assessment and Accreditation of Laboratory Animal Care and were approved by the Institutional Animal Care and Use Committee of Washington State University (protocol number 6031). Male Sprague-Dawley rats weighing between 350–420 g at the time of the experiments were purchased from Envigo. During experiments, animals were individually housed in temperature-controlled (23 ± 1°C), sound-attenuated environmental chambers, operating on a 12:12-hour light-dark cycle (lights on at 3 AM). Humidity was maintained between 30–50%. Rats had unrestricted access to food (Harlan Teklad, Product no. 2016) and water throughout the entire duration of the experiments.

### Materials Used

LPS from Escherichia coli O111 :B4 (dissolved in isotonic NaCl; Sigma-Aldrich), indomethacin (suspended in 1 % Tween 20; Sigma-Aldrich), PGE_2_ (dissolved in Tocrisolve 100 and diluted with isotonic NaCl; Sigma-Aldrich), and arachidonic acid (dissolved in Tocrisolve 100, Tocris) and their respective vehicles were administered in a volume of 1 ml/kg.

### Surgical Procedures

All surgical procedures were performed using ketamine-xylazine anesthesia (87 and 13 mg/kg, respectively, intraperitoneally). Animals received pain management with 0.05% lidocaine administered intradermally, 1 mg/kg buprenorphine ER and 5 mg/kg carprofen, both subcutaneously.

For sleep-wake activity recordings, male Sprague-Dawley rats were implanted with three cortical electroencephalographic (EEG) electrodes, placed over the frontal and parietal cortices, and two electromyographic (EMG) electrodes in the neck muscles. Leads from the EEG and EMG electrodes were anchored to the skull with dental cement. Telemetry transmitters were implanted into the abdominal cavity for body temperature and motor activity measurements. In addition, the rats were implanted with an intraportal catheter three weeks prior to the sleep surgery. Briefly, a biocompatible polyurethane was inserted into the superior mesenteric vein and the tip of the cannula routed to the main stream of the portal vein. The free end of the cannula was routed subcutaneously to the dorsal surface of the neck and exteriorized. The cannula was sutured to the portal vein, the abdominal muscles and the neck skin. In addition to the intraportal cannula, a set of rats were also implanted with an additional cannula into the right jugular vein. Catheter patency was maintained by flushing with 0.2 ml isotonic NaCl followed by 0.08 ml of lock solution containing 500 lU/ml heparin in 50% glycerol solution. The animals were allowed to recover from surgery for at least 10 days before any experimental manipulation started and handled daily to adapt them to the experimental procedures.

### Sleep recordings and analyses

Animals were connected to the recording system through a lightweight, flexible tether plugged into a commutator, which was further routed to Grass Model 15 Neurodata amplifier system (Grass Instrument Division of Astro-Med, Inc., West Warwick, Rl). The amplified EEG and EMG signals were digitized at 256 Hz and recorded by computer. The high-pass and low-pass filters for EEG signals were 0.5 and 30.0 Hz, respectively. The EMG signals were filtered with low and high cut-off frequencies at 100 and 10,000 Hz, respectively. The outputs from the 12A5 amplifiers were fed into an analog-to-digital converter and collected by computer using SleepWave software (Biosoft Studio, Hersey, PA). Sleep-wake states were scored visually off-line in 10-s segments. The vigilance states were defined as non-rapid-eye movement sleep (NREMS), rapid-eye movement sleep (REMS) and wakefulness according to standard criteria as described previously (Szentirmai et al., 2014). Artifact-free EEG epochs were subjected to off-line spectral analysis by fast Fourier transformation. EEG power data in the range of 0.5 to 4.0 Hz during NREMS were used to compute EEG slow-wave activity (SWA). EEG SWA data were normalized for each animal by using the average EEG SWA across 24 h on the baseline day as 100.

### Telemetry recordings of body temperature and motor activity

Core body temperature and locomotor activity were recorded by MiniMitter telemetry system recordings (Starr Life Sciences Corp., model PDT 4000 E-Mitter and ER-4000 receiver) using VitalView software. Temperature and activity values were collected every 1 and 10 min, respectively, throughout the experiment and were averaged over 2-h time blocks.

### Experimental procedures

#### Experiment 1: *The effects of portal vein administration of LPS on sleep, body temperature and motor activity.*

To mimic the translocation of LPS from the intestines into the portal circulation, intraportal injections of LPS were performed in rats. On the baseline day, isotonic NaCl was administered through the cannula. On the test day, 0.1 (n = 5), 1 (n = 8), 5 (n = 8 for sleep recording, n = 6 for body temperature) or 20 μg/kg (n = 11 for sleep recording, n = 10 for body temperature) LPS was administered intraportally, in a volume of 1 ml/kg. The treatments were performed 5–20 min before dark onset. Sleep and telemetry recordings started at dark onset and continued for 23.5 h.

#### Experiment 2: *The effects of LPS administered into the portal vein on LPS levels in the extraportal systemic circulation.*

Eight rats implanted with both intraportal and intrajugular cannulae were used. On the baseline day, the animals were injected with 1 ml/kg NaCl 5–20 min before dark onset through the porta cannula. Blood samples (0.2 ml) were taken from the jugular cannula 5 min after the treatment. Two days later, on the test day, 1 μg/kg LPS was administered intraportally, and jugular blood samples were obtained at the same time point. After a recovery period of 10 days, the experiment was repeated using five of the rats, but sampling time was 120 min after the portal injections. Blood samples were centrifuged, and plasma stored at −80°C. Plasma concentrations of LPS were determined by using Endotoxin ELISA Kit (Aviva Systems Biology) according to the manufacturer’s instructions.

#### Experiment 3: *The effects of systemic administration of LPS on sleep, body temperature and motor activity.*

On the baseline day, the animals were injected ip with isotonic NaCl. On the test day, 20 μg/kg LPS was administered ip 5–10 minutes before dark onset. Sleep and telemetry recordings started at onset of the dark phase and continued for 23.5 h. Sleep was recorded from 6 animals; body temperature was obtained from an additional four and activity from an additional five rats.

#### Experiment 4: *The effects of indomethacin pretreatment on intraportal LPS-induced sleep, body temperature and motor activity.*

On the baseline day, rats (n = 7) were injected with 1% Tween 20 30 min before the intraportal administration of 1 ml/kg NaCl. On the test day, the animals were injected with 10 mg/kg indomethacin subcutaneously, followed by 1 μg/kg LPS intraportally, 30 min later. The intraportal treatments were performed 5–20 min before dark onset. Sleep and telemetry recordings started at dark onset and continued for 23.5 h.

#### Experiment 5: *The effects of portal vein administration of PGE*_*2*_ *and arachidonic acid on sleep, body temperature and motor activity.*

On the baseline day, rats (n = 5) were injected with vehicle intraportally. On the test day, the animals received 300 μg PGE_2_. After a recovery period of 5 days, the same animals received vehicle again, and the following day 600 μg arachidonic acid. All treatments were performed 5–20 min before dark onset. Sleep and telemetry recordings started at dark onset and continued for 23.5 h.

### Statistics

Time spent in wakefulness, NREMS and REMS, as well as body temperature and motor activity, were calculated in 2- and 12-h blocks. The average duration and the total number of sleep and wake episodes were calculated in 12-h blocks.

Paired t-tests were performed on 12-h data between baseline and test days. Two-way repeated measures ANOVA was performed across 24 h between test days and the corresponding baselines on data collapsed in 2-h time blocks (factors: treatment and time, both repeated). When appropriate, Tukey’s Honestly Significant Difference test HSD test was applied post hoc. An α-level of P < 0.05 was considered to be significant.

## Results

### Experiment 1: The effects of portal vein administration of LPS on sleep, body temperature and motor activity.

**Figure 1:**
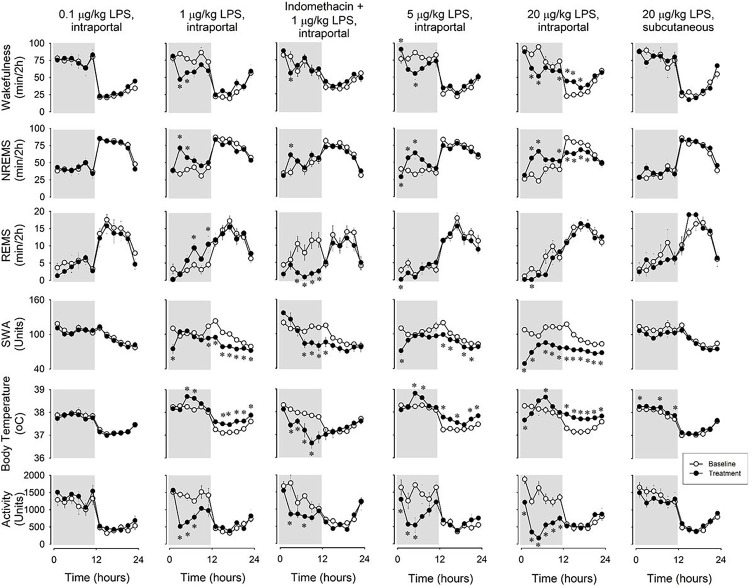
Effects of portal vein administration of 0.1, 1, 5 and 20 μg/kg lipopolysaccharide (LPS), subcutaneous administration of 20 μg/kg LPS and combined indomethacin pretreatment and intraportal administration of 1 μg/kg LPS on wakefulness, non-rapid-eye movement sleep (NREMS), rapid-eye movement sleep (REMS), electroencephalographic slow wave activity (EEG SWA), body temperature and motor activity in rats. Data are expressed in 2-h time blocks. LPS and saline were injected at time “0”. Grey shaded areas represent the dark period; error bar: SE. Asterisks: significant difference from baseline, Tukey’s HSD test.

To assess the influence of portal vein administration of LPS on sleep and body temperature dynamics, a comprehensive dosage range (0.1–20 μg/kg) was tested (Fig. 1, Table 1).

**Figure 2 :**
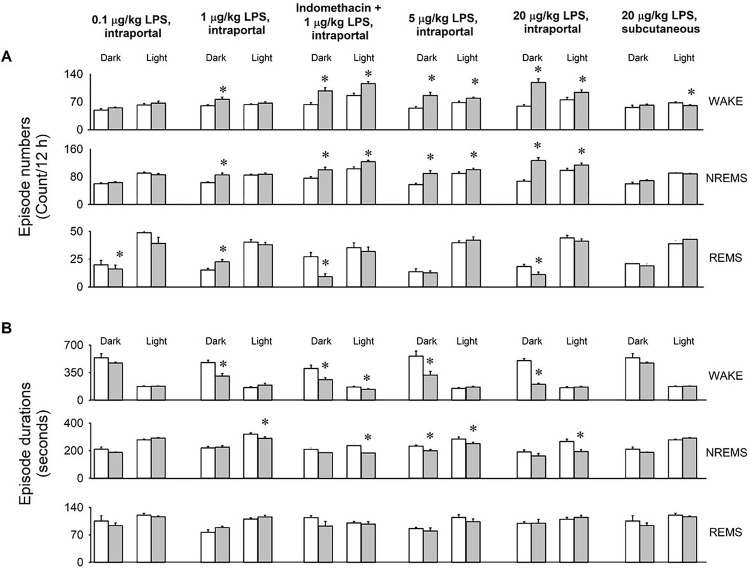
Effects of portal vein administration of 0.1, 1, 5 and 20 μg/kg LPS, subcutaneous administration of 20 μg/kg LPS and combined indomethacin pretreatment and intraportal administration of 1 μg/kg LPS on the number and average duration of wakefulness, NREMS and REMS episodes. Open bars: baseline day, gray bars: treatment day. Data are expressed in 12-h time blocks. Asterisks: significant difference from baseline, Tukey’s HSD test; error bar: SE.

The results revealed robust increases in NREMS duration and decreases in EEG SWA and motor activity, particularly evident during the initial 12 hours after LPS treatments. These effects manifested with a latency of approximately two hours. The minimum effective LPS dose was 1 μg/kg, prompting an approximately 40% increase in NREMS during the dark period (baseline: 228.4 ± 11.6 min vs. LPS: 315.1 ± 15.6 min, p< 0.001, paired t-test). Similar NREMS effects were observed with higher doses. There was a significant, ~ 70%, increase in REMS after 1 μg/kg LPS (baseline: 20.0 ± 3.0 min vs. LPS: 33.6 ± 3.7 min, p < 0.01, paired t-test). Following 20 μg/kg LPS administration, REMS decreased in the dark phase, accompanied by a negative NREMS rebound in the light phase. Sleep architecture became fragmented, evident in increased sleep and wake episode numbers and decreased average bout durations (Fig. 2).

After the injection of 1 and 5 μg/kg LPS, biphasic EEG SWA responses were observed. The initial decrease in the first 2-hour time block, EEG SWA returned to baseline, which was followed by a second, long-lasting suppression. The EEG changes after the highest dose exhibited similar dynamics, however SWA never returned to baseline during the recording period.

Notably, LPS induced prolonged increases in body temperature after an approximately 4-h latency. In response to the highest dose, this effect was preceded by a hypothermic phase.

### Experiment 2: *The effects of LPS administered into the portal vein on LPS levels in the extraportal systemic circulation.*

Intraportal injection of 1 μg/kg LPS did not cause significant changes in LPS levels in the jugular blood. Plasma LPS concentration 5 min after the saline injection was 0.47 ± 0.03 EU/ml, and after LPS injection it was 0.45 ± 0.02 EU/ml (p = 0.64, paired t-test). At 120 minutes, these values were 0.65 ± 0.04 and 0.72 ± 0.04 EU/ml, respectively (p = 0.27, paired t-test). When comparing baselines, the 120-minute samples exhibited significantly higher LPS levels (Student’s t-test, p < 0.01), which likely indicates diurnal changes in circulating LPS levels.

### Experiment 3: The effects of systemic administration of LPS on sleep, body temperature and motor activity.

To demonstrate the heightened sensitivity of the hepatoportal region to LPS, we assessed the effects of extraportal systemic LPS administration, using a dose known to induce sleep when delivered intraportally (20 μg/kg LPS, subcutaneously). Notably, this treatment did not yield any significant effects on sleep-wake states, EEG SWA, or motor activity (Μg. 1). ANOVA revealed a minor, but statistically significant treatment effect for body temperature (Table 2). Average body temperature on the baseline day was 37.58°C, while after LPS treatment it showed a modest increase to 37.69°C — an elevation of 0.11°C over the 24-h period.

### Experiment 4: The effects of indomethacin pretreatment on intraportal LPS-induced increased sleep and body temperature and suppressed motor activity.

To assess the role of prostaglandins in the effects of intraportally delivered LPS, we pretreated the animals with indomethacin, an inhibitor of prostaglandin synthesis, prior to the injection of 1 μg/kg LPS. Indomethacin significantly suppressed the NREMS-promoting actions of LPS, and completely blocked LPS-induced body temperature and REMS increases (Fig. 1). ANOVA revealed a significant treatment x time interaction for NREMS in the pretreated animals (see Table 3), albeit the effects were significantly attenuated when compared to the effects of LPS without indomethacin pretreatment. Without indomethacin, LPS induced an 86.7 ± 15.6 min increase in NREMS amounts during the 12-hour dark period. In contrast, after indomethacin pretreatment, the increase was only 38.0 ± 11.4 min, a significant difference in the effects of LPS (p< 0.05).

Indomethacin abolished the immediate EEG SWA suppression observed in the first 2-h time period after 1 μg/kg LPS. However, the late suppression of EEG SWA during the second 12-hour period after treatment remained unaffected.

Without pretreatment, 1 μg/kg LPS led to increases in REMS and body temperature. Remarkably, indomethacin pretreatment transformed both responses into profound suppression. REMS was nearly completely eliminated for 12 hours after LPS administration, returning to baseline levels in the second half of the recording period. Body temperature decreased throughout the dark period, reaching a hypothermia of 1.2°C 10 hours after LPS injection. The decreased motor activity response to LPS was not affected by indomethacin pretreatment.

### Experiment 5: The effects of portal vein administration of PGE_2_ and arachidonic acid (AA) on sleep, body temperature and motor activity.

**Figure 3 :**
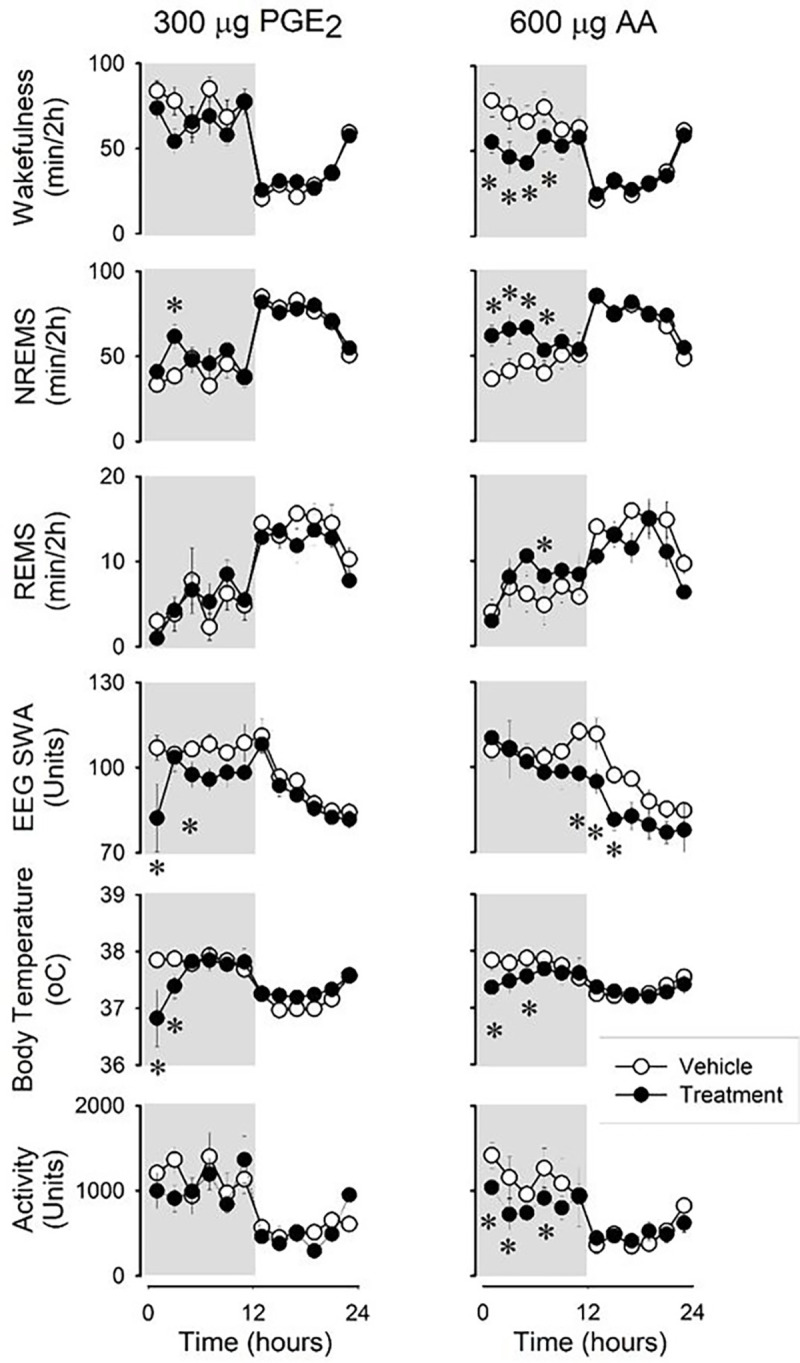
Effects of portal vein administration of prostaglandin E_2_ (PGE_2_) and arachidonic acid (AA) on wakefulness, NREMS, REMS, EEG SWA, body temperature and motor activity in rats. See legends to Figure 1 for details.

**Figure 4 :**
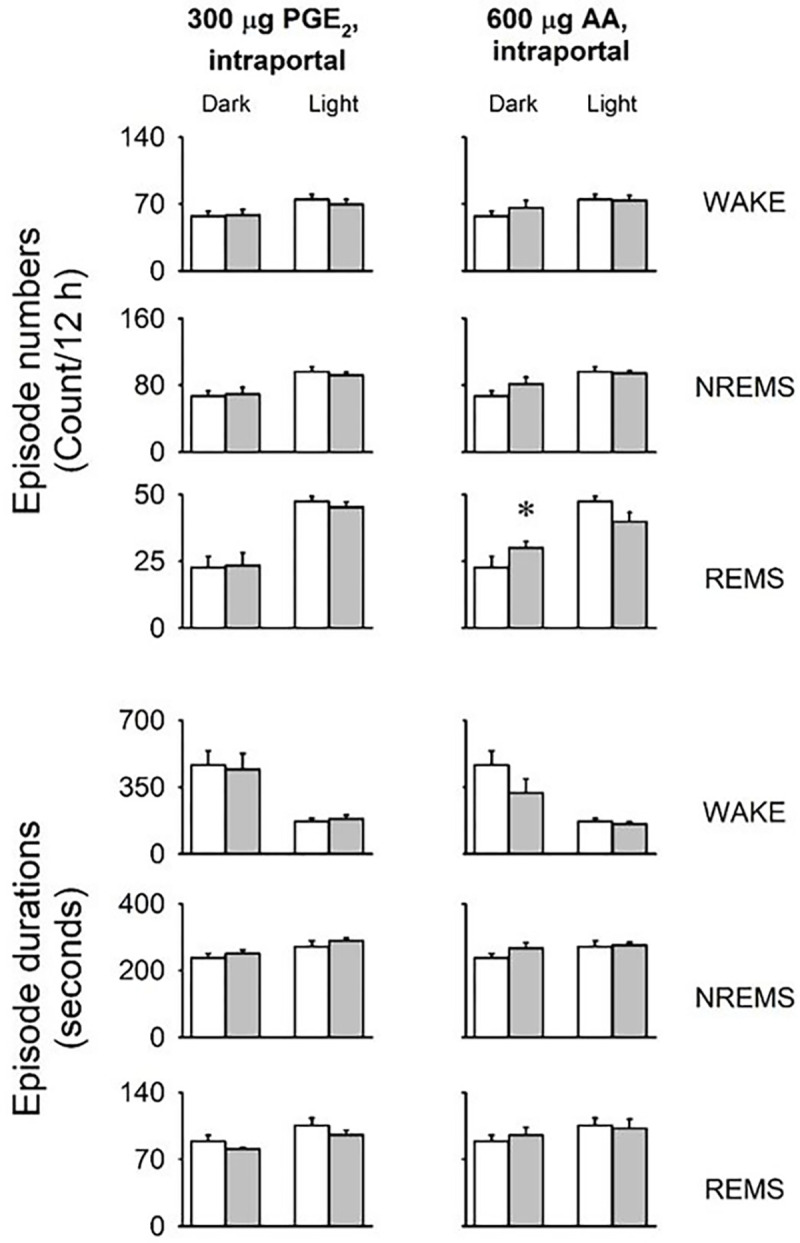
Effects of portal vein administration of PGE_2_ and arachidonic acid on the number and average duration of wakefulness, NREMS and REMS episodes. See legends to Figure 2 for details.

We examined whether the intraportal administration of PGE_2_ and the prostaglandin precursor AA could replicate the effects observed after LPS. Over the 12-hour period following the injections, both PGE_2_ and AA induced significant increases in NREMS and reductions in body temperature (see Fig. 3, Table 4). In the dark phase, PGE_2_ and AA induced approximately a 22% and 36% rise in NREMS, respectively (PGE_2_ baseline: 236.2 ± 31.0 min, treatment: 287.1 ± 29.5 min, p < 0.01; AA baseline: 264.6 ± 30.5 min, treatment: 358.6 ± 34.0 min, p< 0.05). Additionally, there was a trend toward increased REMS in response to AA (baseline: 34.7 ± 7.9 min, treatment: 47.1 ± 4.1 min, p = 0.057). The number and average durations of vigilance states were not affected by the treatments with the exception of increased REMS episode numbers in the dark phase after AA administration (Fig. 4).

PGE_2_ exhibited a suppressive effect on EEG SWA, and during the light phase, an increase in body temperature was observed. While there were tendencies toward decreased motor activity, neither PGE_2_ nor AA administration led to statistically significant changes.

## Discussion

Growing body of evidence supports the notion that the microbiota serves as a source of sleep-promoting signals (Brown et al., 1990; Millican et al., 2018; Ogawa et al., 2020, Szentirmai et al., 2019; Szentirmai et al., 2021). We hypothesized that LPS, a component of gram-negative bacterial cell wall, could function as one such sleep-inducing signal. Our main finding is that LPS induces increased sleep and elevated body temperature when injected into the portal circulation. The portal circulation drains blood from organs housing the intestinal microbiota, including the stomach, small intestine, and large intestine.

Microbial molecules, such as LPS, enter the portal circulation, thus the internal environment of the host, by the process called translocation. The free translocation of microbial molecules is prevented by the intestinal barrier, nevertheless, significant amounts of these molecules appear in the portal blood under normal healthy conditions (Guerville and Boudry, 2016). The translocation of LPS from the intestinal lumen to the portal blood is facilitated by transport mechanisms associated with the LPS receptor TLR4 (Cetin et al., 2004). LPS is naturally present in both portal and extraportal systemic blood in healthy humans, rats, and mice, rendering it a physiological plasma component of prokaryotic origin (Nolan, 1981; Wiedermann et al., 1999; Goto et al., 1994). Everyday stimuli further facilitate translocation, consequently elevating circulating levels of LPS. For example, a single high-fat meal consisting of three slices of toast spread with 50 g butter elevates plasma LPS levels by 50% in humans (Erridge et al., 2007). Additionally, common occurrences such as consumption of relatively low amounts of alcohol (Ferrier et al., 2006; Sturm et al., 2021), acute or chronic sleep loss (Wang et al., 2021; Li et al., 2024) and mild psychological stress (Vanuytsel et al., 2013; Santos et al., 2000) all contribute to increased circulating levels of LPS.

Given the pervasive presence of LPS in circulatory systems and the observation that systemic administration of high, proinflammatory doses of LPS induces sleep in various species, including humans (Lancel et al., 1995; Kapas et al., 1998; Opp and Toth, 1998; Pollmacher et al., 1993), we postulated that LPS translocated into the portal circulation might serve as a signal from the intestinal microbiota to initiate sleep. If proven valid, we anticipated that mimicking increased LPS translocation into the portal circulation would enhance sleep. Our findings support this hypothesis, revealing that even a minimal dose of 1 μg/kg LPS injected into the portal vein induces increases in NREMS and elevated body temperature.

Multiple lines of evidence suggest that the effects of intraportally injected LPS are not systemic but rather arise from the activation of LPS receptors located in the portal circulation or within the liver. First, our previous work has identified the existence of a sleep-inducing sensory mechanism in the hepatoportal region, sensitive to microbial molecules such as the short-chain fatty acid butyrate and LTA, a cell wall component of gram-positive bacteria (Szentirmai et al., 2019; Szentirmai et al., 2021). Second, systemic administration of LPS at a dose 20 times higher than the lowest effective intraportal dose failed to impact sleep and did not elicit any biologically meaningful effect on body temperature. Moreover, systemic administration of the lowest effective dose of LPS following intraportal administration failed to induce changes in LPS levels in the extraportal systemic circulation. This observation is consistent with the concept that the liver functions as an effective LPS sink. Notably, 1 g liver tissue has the capacity to clear intraportally administered LPS at the rate of 1.5 μg/h during a single passage (Yamaguchi et al., 1982). Considering that the liver weight of a 400 g rat is approximately 14 g (Webster et al., 1947), the liver can remove 21 μg of LPS in an hour or 0.35 μg in one minute. In our experiment, we injected 1 μg/kg (equivalent to approximately 0.4 μg/animal LPS) over a period of two minutes. These theoretical considerations robustly support the notion that low doses of LPS, when directly injected into the portal vein, undergo significant clearance by the liver and do not result in overflow into the hepatic vein.

Several potential cellular targets for LPS exist in the hepatoportal region. The LPS receptor, TLR4, is expressed by various liver cells, including hepatic macrophages (Kupffer cells), sinusoidal endothelial cells, stellate cells, and hepatocytes (Nakamoto and Kanai, 2014). Additionally, the liver and the portal vein wall receive innervation from vagal afferents, which also express TLR4 receptors (Berthoud et al., 1992; Hosoi et al., 2005; Kunda et al., 2014). Given that the sleep-promoting and febrile responses to LPS manifest after a latency of 2–4 hours, it is unlikely that these effects result from the direct activation of sensory nerves by LPS. Instead, it suggests that the effects involve the activation of a slower biochemical machinery leading to the production of other molecular mediators in the liver. Among the liver cell types, Kupffer cells are the most extensively studied targets for LPS. In response to LPS, Kupffer cells produce molecules known to affect sleep, including proinflammatory cytokines such as interleukin (IL)-6 and tumor necrosis factor-α(TNFα), along with PGE2, (Nakamoto and Kanai, 2014; Bowers et al., 1985; Peters et al., 1990).

Our unpublished preliminary data, demonstrating the complete abolition of the sleep-inducing effects of systemic LPS in cyclooxygenase (COX)-2 knockout mice, prompted further investigation into the involvement of prostaglandins in mediating the effects of intraportally administered LPS. Notably, pretreatment with the COX inhibitor indomethacin significantly suppressed the sleep-inducing actions of LPS, underscoring the role of prostaglandins in mediating these effects. The findings that intraportal injections of PGE_2_ and the prostaglandin precursor AA also exhibit sleep-promoting effects indirectly support the involvement of hepatic prostaglandin production in LPS-induced sleep. Peripherally produced prostaglandins have been demonstrated to possess sleep-promoting properties. Nicotinic acid, a potent stimulant for prostaglandin synthesis in keratinocytes and Langerhans cells of the skin, exhibits robust sleep-promoting effects. Research has shown that sleep induced by nicotinic acid is abolished by indomethacin (Szentirmai and Kapas, 2019).

The role of prostaglandins in LPS-induced sleep has been previously investigated in mice (Oishi et al., 2015). In that study, somnogenic effects of high doses (~ 100 μg/kg) of systemically administered LPS were not blocked by the COX inhibitor meloxicam, and the effects were not attenuated in mice lacking EP3 receptors in the nervous system or mice with a total body KO of microsomal PGE synthase-1 or the PGD2 receptor type DP. There was a slight reduction in the sleep-inducing effects in mice with a nervous system-specific knockout of the EP4 receptor. It is important, however, to emphasize the differences between our model, where a low dose of LPS was injected intraportally and did not elicit changes in systemic LPS levels, and the above mouse model, where high doses of LPS were injected systemically, likely reaching central and other peripheral targets. Although LPS does not cross the blood-brain barrier (Banks and Robinson, 2010), it can still act on central sites, such as endothelial cells of the cerebral vessels, or neurons and glia at sites where the blood-brain barrier is incomplete and permeable to LPS (Matsumura et al., 1998; Blatteis et al., 1983). When LPS reaches brain target sites, it can induce fever and sleep (Krueger et al., 1986; Zielinski et al., 2017; Steiner and Branco, 2000). Furthermore, the sleep-promoting actions of high doses of systemically administered LPS are also attributed to uncoupling protein-1-dependent thermogenic mechanisms of the brown adipose tissue (Szentirmai and Kapas, 2018). These central and extrahepatic peripheral sleep-promoting effects of LPS may occur independently of the prostaglandin system.

Increased REMS observed after lower doses of LPS, and the REMS suppression following the highest intraportal dose, align with the notion that increasing doses of systemically administered LPS trigger REMS suppression (Szentirmai and Krueger, 2014). The observation that indomethacin pretreatment suppressed both body temperature and REMS, coupled with the finding that high LPS doses also suppress both body temperature and REMS, suggests a potential relationship between reduced REMS and body temperature. This is consistent with the concept that the actual thermoregulatory changes themselves may play a role in sleep regulation (Gilbert et al., 2004).

It has been proposed that hepatic prostaglandin production is a key factor in fever induced by systemic LPS administration (Roth and Blatteis, 2014). Our findings are consistent with this notion. Our results not only demonstrate an increase in sleep but also reveal an elevation in body temperature following the lowest dose of intraportally injected LPS, strongly suggesting a hepatic site of action for LPS-induced fever. The observed 4-h latency to increased body temperature may be attributed to the de novo production of a febrile mediator. The complete abolition of fever by indomethacin underscores the critical role of prostaglandins in this response.

Moreover, indomethacin pretreatment not only completely prevented LPS-induced fever but, intriguingly, transformed the response into profound hypothermia. Similar hypothermic responses to LPS have been reported in other models with suppressed prostaglandin production or in mice deficient in IL-6, IL-10, and NF-κB (Kozak et al., 1994; Zhang et al., 2003; Toth and Opp, 2001; Morrow and Opp, 2005; Jhaveri et al., 2006). These collective findings support the notion that LPS has two independent effects on body temperature (Dogan et al., 2002). It promotes fever through the activation of the proinflammatory IL-6/IL-10 - PGE2 - NF-κB axis and, independently, it induces hypothermia. The activation of the proinflammatory arm likely masks the hypothermic effects, but inhibiting the proinflammatory process unveils hypothermia. Furthermore, higher doses of systemic LPS exhibit biphasic effects on body temperature, with fever preceded by an initial hypothermic phase. This dual action of LPS on body temperature may explain the observed biphasic response. Additionally, beyond the “classic” LPS receptor TLR4, LPS also binds to and activates transient receptor potential A1 (TRPA1) receptors on vagal afferents (Meseguer et al., 2014). Transsection of the subdiaphragmatic vagal trunk abolishes the hypothermic effect of high doses of LPS (Kapas et al., 1998), while the activation of vagal TRPA1 receptors by IL-1 or 2-methyl-2-thiazoline induces hypothermia (Matsuo et al., 2021; Silverman et al., 2023). Therefore, it is possible that the hypothermic actions of LPS are mediated through the activation of vagal TRPA1 receptors.

EEG SWA reflects the prevalence of delta waves during NREMS. EEG SWA is influenced by sleep pressure (Borbely et al., 2016), and can also change independently of sleep-wake activity in response to various drugs such as atropine (Bradley, 1968), metabolic alterations (Lewis et al., 1974), or the activity of thermoregulatory mechanisms (Berger et al., 1998; Gaenshirt et al., 1954). The biphasic SWA responses after low-dose intraportal LPS treatment were similar to those observed in response to 100 μg/kg LPS given ip (Kapas et al., 1998). It remains unclear whether the LPS-induced suppression of EEG SWA reflects reduced NREMS pressure or if it represents a sleep-independent effect on cortical neuronal activity. In either scenario, the effect likely originates from the hepatoportal region, as evidenced by the lack of effect after extraportal systemic LPS treatment. Indomethacin abolished the first phase of EEG SWA suppression, suggesting the involvement of prostaglandins. Previously, we reported that subdiaphragmatic vagotomy abolishes the effect of systemic LPS treatment on EEG SWA (Kapas et al., 1998). Furthermore, vagal stimulation has a profound effect on EEG activity (Grastyan et al., 1952; Bonvallet and Sigg, 1958; Chase et al., 1967). These observations are consistent with the notion that the EEG effects of LPS may be mediated, at least in part, by vagus afferents in response to prostaglandin production in the liver.

Indomethacin pretreatment did not completely prevent LPS-induced sleep, suggesting that besides prostaglandins, other mediators may also play a role in the effects of LPS. Kupffer cells, stellate cells, and sinusoidal endothelial cells produce TNFα and IL-6 in response to LPS (Seki et al., 2001; Thirunavukkarasu et al., 2005). Both cytokines have NREMS-promoting actions, potentially contributing to the effects of LPS (Kapas et al., 1992; Szentirmai and Kapas, 2019; Hogan et al., 2003). These mediators may be released into the hepatic vein and reach distant targets through the systemic circulation. Alternatively, they could act locally on afferent neurons, as both the vagus and spinal nerves provide afferent innervation to the liver. Vagal afferents express the PGE2 receptors EP3 and EP4 as well as TNFα p55 receptors (Hermann et al., 2005; Steinberg et al., 2016; Ek et al., 1998). Both PGE2 and TNFα stimulate afferent vagal activity (Birrell et al., 2013; Hermann et al., 2005). It is possible that locally produced PGE2, and possibly other mediators, stimulate vagal afferents to send somnogenic signals to brain core sleep circuits. This aligns with findings indicating that the vagus nerve carries sleep-inducing input to the brain (Armitage et al., 2003; Bonvallet and Sigg, 1958), and the sleep-promoting and fever-inducing actions of systemically administered high doses of LPS are suppressed by vagal nerve transection (Kapas et al., 1998; Opp and Toth, 1998).

In summary, our findings strongly support the idea that LPS, translocated from the intestinal lumen into the portal circulation, plays a role in modulating sleep and body temperature. We propose that, in conjunction with other bacterial metabolites such as butyrate and LTA, and along with other potential microbial molecules, LPS contributes to a complex molecular signaling repertoire targeting the liver to activate hepatoportal sensors. The signals arising from these sensors likely traverse to central sleep circuits, utilizing either neuronal or humoral pathways. This multifaceted molecular interplay underscores the intricate connections between the gut microbiota, microbial molecules and the regulation of sleep-wake cycles.

## Figures and Tables

**Table 1.** Intra-portal administration of lipopolysaccharide (LPS). Non-rapid eye movement sleep (NREMS), rapid-eye movement sleep (REMS), body temperature, motor activity and electroencephalographic slow-wave activity (SWA): statistical results

*0.1 mg/kg LPS, intraportally*																		
	Wakefulness			NREMS			REMS			Temperature			Activity			SWA		
	*df*	*F*	*p*	*df*	*F*	*p*	*df*	*F*	*p*	*df*	*F*	*p*	*df*	*F*	*p*	*df*	*F*	*p*
*Treatment*	1,4	4.2	n.s.	1,4	2.8	n.s.	1,4	4.6	n.s.	1,4	12.0	<0.05	1,4	34.5	<0.01	1,4	0.2	n.s.
*Time*	11,44	23.6	<0.001	11,44	22.8	<0.001	11,44	11.6	<0.001	11,44	15.1	<0.001	11,44	22.2	<0.001	11,44	18.5	<0.001
*Treatment × Time*	11,44	0.6	n.s.	11,44	0.6	n.s.	11,44	0.6	n.s.	11,44	0.4	n.s.	11,44	0.6	n.s.	11,44	1.1	n.s.
*1 mg/kg LPS, intraportally*																		
	Wakefulness			NREMS			REMS			Temperature			Activity			SWA		
	*df*	*F*	*p*	*df*	*F*	*p*	*df*	*F*	*p*	*df*	*F*	*p*	*df*	*F*	*p*	*df*	*F*	*p*
*Treatment*	1,7	36.1	<0.001	1,7	26.9	<0.01	1,7	6.8	<0.05	1,7	60.2	<0.001	1,7	22.1	<0.01	1,7	40.5	<0.001
*Time*	11,77	30.1	<0.001	11,77	28.9	<0.001	11,77	21.0	<0.001	11,77	80.0	<0.001	11,77	26.9	<0.001	11,77	16.4	<0.001
*Treatment × Time*	11,77	5.1	<0.001	11,77	5.8	<0.001	11,77	30.5	<0.05	11,77	4.7	<0.001	11,77	6.4	<0.001	11,77	8.3	<0.001
*5 mg/kg LPS, intraportally*																		
	Wakefulness			NREMS			REMS			Temperature			Activity			SWA		
	*Df*	*F*	*p*	*df*	*F*	*p*	*df*	*F*	*p*	*df*	*F*	*p*	*df*	*F*	*p*	*df*	*F*	*p*
*Treatment*	1,7	2.4	n.s.	1,7	4.5	n.s.	1,7	7.8	<0.05	1,5	8.5	<0.05	1,7	9.0	<0.05	1,7	11.7	<0.05
*Time*	11,77	33.2	<0.001	11,77	26.6	<0.001	11,77	35.2	<0.001	11,55	48.6	<0.001	11,77	12.4	<0.001	11,77	15.2	<0.001
*Treatment × Time*	11,77	4.3	<0.001	11,77	5.1	<0.001	11,77	1.2	n.s.	11,55	4.0	<0.001	11,77	6.1	<0.001	11,77	3.6	<0.001
*20 mg/kg LPS, intraportally*																		
	Wakefulness			NREMS			REMS			Temperature			Activity			SWA		
	*df*	*F*	*p*	*df*	*F*	*p*	*df*	*F*	*p*	*df*	*F*	*p*	*df*	*F*	*P*	*df*	*F*	*p*
*Treatment*	1,10	2.1	n.s.	1,10	5.7	<0.05	1,10	6.6	<0.05	1,9	11.5	<0.01	1,9	187.8	<0.001	1,10	47.3	<0.001
*Time*	11,110	40.2	<0.001	11,110	34.7	<0.001	11,110	37.4	<0.001	11,99	33.2	<0.001	11,99	18.9	<0.001	11,110	19.4	<0.001
*Treatment × Time*	11,110	12.5	<0.001	11,110	18.3	<0.001	11,110	1.7	n.s.	11,99	7.7	<0.001	11,99	12.7	<0.001	11,110	11.8	<0.001

**Table 2:** Subcutaneous administration of LPS. NREMS, REMS, body temperature, motor activity and EEG SWA: statistical results **20 μg/kg LPS, subcutaneously**

	Wakefulness			NREMS			REMS			Temperature			Activity			SWA		
	*df*	*F*	*p*	*df*	*F*	*p*	*df*	*F*	*p*	*df*	*F*	*p*	*df*	*F*	*P*	*df*	*F*	*p*
*Treatment*	1,5	0.1	n.s.	1,5	2.0	n.s.	1,5	0.0	n.s.	1,9	18.0	< 0.01	1,10	1.2	n.s.	1,5	2.4	n.s.
*Time*	11,55	25.1	< 0.001	11,55	28.0	< 0.001	11,55	9.0	< 0.001	11,99	76.0	< 0.001	11,110	28.4	< 0.001	11,55	12.5	< 0.001
*Treatment × Time*	11,55	1.8	n.s.	11,55	1.4	n.s.	11,55	1.9	n.s.	11,99	1.0	n.s.	11,110	1.4	n.s.	11,55	0.8	n.s.

**Table 3:** Indomethacin pretreatment + intra-portal administration of 1 μg/kg LPS. NREMS, REMS, body temperature, motor activity and EEG SWA: statistical results **Indomethacin + 1 μg/kg LPS, intraportally**

	Wakefulness			NREMS			REMS			Temperature			Activity			SWA		
	*df*	*F*	*p*	*df*	*F*	*p*	*df*	*F*	*p*	*df*	*F*	*p*	*df*	*F*	*P*	*df*	*F*	*p*
*Treatment*	1,6	0.9	n.s.	1,6	0.6	n.s.	1,6	101	< 0.05	1,6	9.0	< 0.05	1,6	21.4	< 0.01	1,6	2.3	n.s.
*Time*	11,66	16.8	< 0.001	11,66	22.2	< 0.001	11,66	7.7	< 0.001	11,66	11.0	< 0.001	11,66	11.9	< 0.001	11,66	13.6	< 0.001
*Treatment × Time*	11,66	1.9	< 0.05	11,66	2.7	< 0.01	11,66	2.6	< 0.01	11,66	6.0	< 0.001	11,66	2.9	< 0.01	11,66	4.8	< 0.001

**Table 4:** Intra-portal administration of prostaglandin E_2_ (PGE_2_) and arachidonic acid (AA). NREMS, REMS, body temperature, motor activity and EEG SWA: statistical results **300 μg PGE2, intraportally**

	Wakefulness			NREMS			REMS			Temperature			Activity			SWA		
	*df*	*F*	*p*	*df*	*F*	*p*	*df*	*F*	*p*	*df*	*F*	*p*	*df*	*F*	*P*	*df*	*F*	*p*
*Treatment*	1,4	13.9	< 0.05	1,4	12.1	< 0.05	1,4	17.3	< 0.05	1,4	0.5	n.s.	1,4	2.0	n.s.	1,4	9.1	< 0.05
*Time*	11,44	26.8	< 0.001	11,44	27.5	< 0.001	11,44	8.9	< 0.001	11,44	7.5	< 0.001	11,44	12.0	< 0.001	11,44	7.3	< 0.001
*Treatment × Time*	11,44	6.2	< 0.001	11,44	5.9	< 0.001	11,44	4.2	< 0.001	11,44	4.9	< 0.001	11,44	1.8	n.s.	11,44	1.4	n.s.
600 μg AA, intraportally																		
	Wakefulness			NREMS			REMS			Temperature			Activity			SWA		
	*df*	*F*	*p*	*df*	*F*	*p*	*df*	*F*	*p*	*df*	*F*	*p*	*df*	*F*	*P*	*df*	*F*	*p*
*Treatment*	1,4	23.4	< 0.01	1,4	22.1	< 0.01	1,4	17.3	< 0.05	1,4	1.0	n.s.	1,4	4.2	n.s.	1,4	2.0	n.s.
*Time*	11,44	10.7	< 0.001	11,44	10.1	< 0.001	11,44	8.9	< 0.001	11,44	4.1	< 0.001	11,44	7.3	< 0.001	11,44	10.6	< 0.001
*Treatment × Time*	11,44	2.4	< 0.05	11,44	2.4	< 0.05	11,44	4.2	< 0.001	11,44	3.4	< 0.01	11,44	1.8	n.s.	11,44	2.1	< 0.05

## Data Availability

The data that support the findings of this study are available from the corresponding author upon reasonable request.
